# Anteroposterior Translation Does Not Correlate With Knee Flexion After Total Knee Arthroplasty

**DOI:** 10.1007/s11999-013-3274-2

**Published:** 2013-09-05

**Authors:** Yoshinori Ishii, Hideo Noguchi, Mitsuhiro Takeda, Junko Sato, Shin-ichi Toyabe

**Affiliations:** 1 Ishii Orthopaedic and Rehabilitation Clinic, 1089 Shimo-Oshi, Gyoda, Saitama 361-0037 Japan; 2 Division of Information Science and Biostatistics, Niigata University Graduate School of Medical and Dental Sciences, Niigata, Japan

## Abstract

**Background:**

Stiffness after a TKA can cause patient dissatisfaction and diminished function, therefore it is important to characterize predictors of ROM after TKA. Studies of AP translation in conscious individuals disagree whether AP translation affects maximum knee flexion angle after implantation of a highly congruent sphere and trough geometry PCL-substituting prosthesis in a TKA.

**Questions/purposes:**

We investigated whether AP translation correlated with maximum knee flexion angle (1) in patients who were awake, and (2) who were under anesthesia (to minimize the effects of voluntary muscle contraction) in a TKA with implantation of a PCL-substituting mobile-bearing prosthesis.

**Methods:**

AP translation was examined under both conditions in 34 primary TKAs. Measurements under anesthesia were performed when the patients were having anesthesia for a contralateral TKA. Awake measurements were made within 4 days of that anesthetic session in patients who had no residual sedative effects. The average postoperative interval for the index TKA flexion measurements was 23 months (range, 6–114 months). AP translation was evaluated at 75° flexion using an arthrometer.

**Results:**

There was no correlation between postoperative maximum knee flexion and AP translation at 75° during consciousness. There was no correlation between postoperative maximum knee flexion and AP translation under anesthesia.

**Conclusion:**

AP translation at 75° flexion did not correlate with postoperative maximum knee flexion in either awake or anesthetized patients during a TKA with implantation of a posterior cruciate-substituting prosthesis.

**Level of Evidence:**

Level II, therapeutic study. See the Instructions for Authors for a complete description of levels of evidence.

## Introduction

In principle, proper AP translation after TKA facilitates physiologic rollback or posterior slide of the femoral component. PCL-substituting devices prevent early tibiofemoral impingement attributable to excessive pathologic roll-forward or anterior slide. Thus, better knee flexion would be expected with proper AP translation after TKA. The topic is important because, as illustrated by a recent report [12], better ROM is associated with increased patient satisfaction. Knee stability after TKA also is known to be an important driver of patient-specific outcomes after reconstruction and is associated with correct AP translation [4, 6, 10, 13, 14, 16, 24–26].

However, previous clinical studies [3, 8, 10, 21, 24, 26] disagreed regarding whether AP translation is associated with improved knee flexion after TKA. Some studies [8, 10, 21, 24] showed significant correlation, whereas others [3, 25] showed no correlation. Because these studies were performed with patients who were fully conscious, muscle contractions could influence the clinical testing results, and the voluntary and involuntary contractions in that setting would be difficult or impossible to quantify, perhaps causing the differences in the results of the studies.

In the current study, we sought to determine whether AP translation correlated with maximum knee flexion angle in patients who were (1) awake, and (2) under anesthesia (to minimize the effects of voluntary muscle contraction) in a TKA with implantation of a PCL-substituting mobile-bearing prosthesis.

## Patients and Methods

This was a prospective, comparative study. Informed consent, which included a description of the protocol and potential arthrometer-related complications, was obtained from all patients. We received institutional review board approval. All patients received low-contact stress (LCS) prostheses (PCL-substituting, rotating-platform design); 34 knees in 34 patients were evaluated. The LCS prosthesis was constrained in the AP axis and unconstrained in the rotational axis [22]. In the current system, there was full contact between the femoral component and the tibial insert from 0° to 30° knee flexion, and the geometry of the prosthesis involved a progressive posterior decrease in the radius of curvature of the femoral condyle and a decrease in the constraint with flexion between the tibial and femoral components [17].

This study was performed when the patients were readmitted to the hospital for a contralateral knee arthroplasty. The TKAs being evaluated were performed at a mean of 23 months after the index TKA (range, 6–114 months). One surgeon (YI) performed all the TKAs using a standardized technique, including the necessary soft tissue release for proper balance; the surgical technique and rehabilitation protocol are described in detail in a previous report [7]. In all knees, the femoral components were fixed without cement and the tibial components were fixed with cement. Proper intraoperative AP stability was confirmed manually, although it was not quantified intraoperatively. All of the TKAs were judged to be clinically successful (Hospital for Special Surgery scores greater than 90) [1], with no ligamentous instability or pain at the time of measurement. Contraindications for surgery were revision arthroplasties, previous tibial osteotomies, or rheumatoid arthritis. The clinical characteristics of the patients are summarized (Table 1).

**Table 1:** Patient demographics

Variable	PCL-substituting prosthesis
Number of knees/patients	34/34
Sex (male/female)	4/30
Age (years)*	72 (10)
Preoperative median flexion (°) (25^th^ percentile, 75^th^ percentile)	120° (100°, 130°)
BMI (kg/m^2^)*	27 (4)
Hospital for Special Surgery score (points)*^,#^	91 (2)
Posterior slope (°)*^,#^	10 (2)
Coronal alignment (°)*^,§^	6 (3)

Performed using Knee Society radiographic assessment [5]; * values are expressed as mean with SD in parentheses; ^#^evaluated using radiographs; ^§^valgus.

**Fig. 1A–B:**
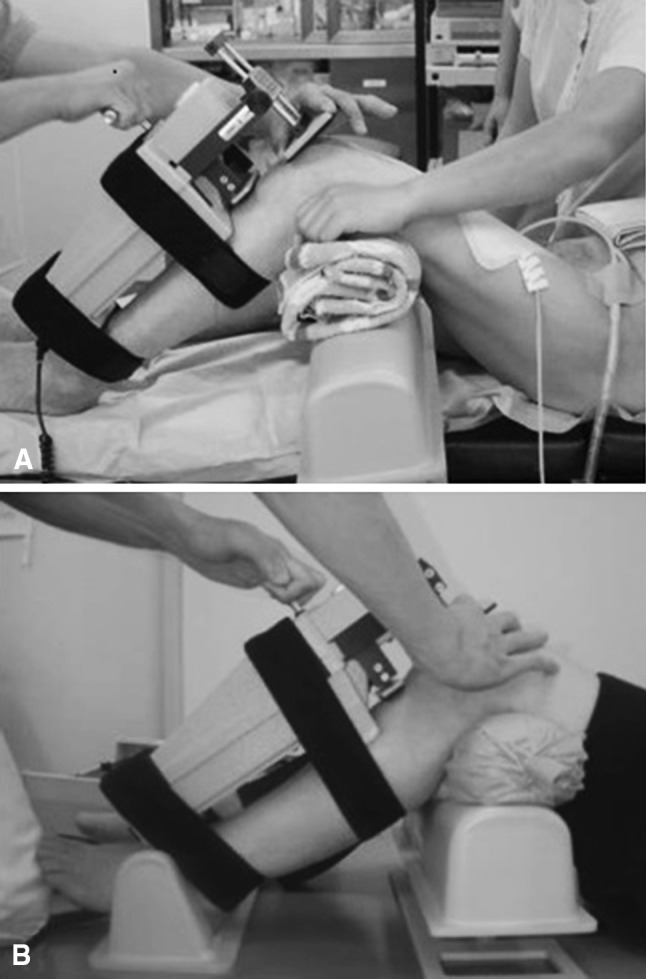
AP translation was measured with a KT-2000 arthrometer using standard protocols. The relative movement between the patellar and tibial tubercle sensor pads was recorded at 75° flexion when the patient was (**A**) under anesthesia and (**B**) while awake when applying an anterior force of 133 N and a posterior force of 89 N.

For this study, each knee was evaluated twice, once when the patient was under anesthesia and a second time while the patient was awake. The evaluation under anesthesia was performed when the patients were having anesthesia for the new contralateral TKA (Fig. 1A). The awake evaluation was performed at a mean of 3 days (range, 2–4 days) after that surgery (Fig. 1B), and when patients were determined not to be under any residual effects of anesthesia, sedation, or regional block.

AP translation was evaluated at 75° flexion, confirmed with a goniometer using a KT2000™ arthrometer (MEDmetric Corp, San Diego, CA, USA). An anterior force of 133 N [6, 13, 16, 26] and a posterior force of 89 N [6, 10, 13, 16] were applied. During the awake evaluations, all patients were observed to relax their quadriceps and hamstrings to minimize voluntary muscular defense. The same observer (TS) performed all tests to eliminate interobserver variation. Three measurements of total AP translation were made and the average value of the three measurements was used. Total AP translation was measured because the position of the femoral component in relation to the tibial component at 75° varied. Intrasubject errors were 0.71 mm (SD, 0.79 mm; range, 0.2–4.7 mm) during consciousness and 0.49 mm (SD, 0.31 mm; range, 0.0–1.2 mm) under anesthesia.

The surgeon (YI) measured maximum knee flexion and extension using a standard hand-held goniometer with 38-cm-long arms while the patient was supine under nonweightbearing conditions. The lateral femoral condyle was used as the landmark to center the goniometer, with the stationary arm directed toward the greater trochanter and the movable arm directed toward the lateral malleolus. The amount of knee flexion was measured and recorded to the nearest 5°.

### Statistical Analysis

Spearman’s rank correlation coefficients were used to evaluate the relationships between AP translation in each condition and the maximum flexion after TKA. We also used Spearman’s rank correlation coefficients to analyze the individual correlations between AP translations in each condition. Based on a one-sided power analysis, we determined 34 samples would be sufficient to detect a correlation coefficient of 0.5 with 92.4% power. The strength of the correlation of rank coefficients was defined as: strong = 0.70–1.0, moderate = 0.40–0.69, or weak = 0.20–0.39. Additionally, the Wilcoxon rank-sum test was used to compare AP translation between the measurements made in awake and anesthetized patients and the maximum knee flexion before and after TKA. All values were expressed using median (25th percentile, 75th percentile). Statistical analyses were performed using SPSS^®^ Version 14.0 J software (SPSS Japan, Inc, Tokyo, Japan). In all tests, p values less than 0.05 were considered statistically significant.

## Results

**Fig. 2:**
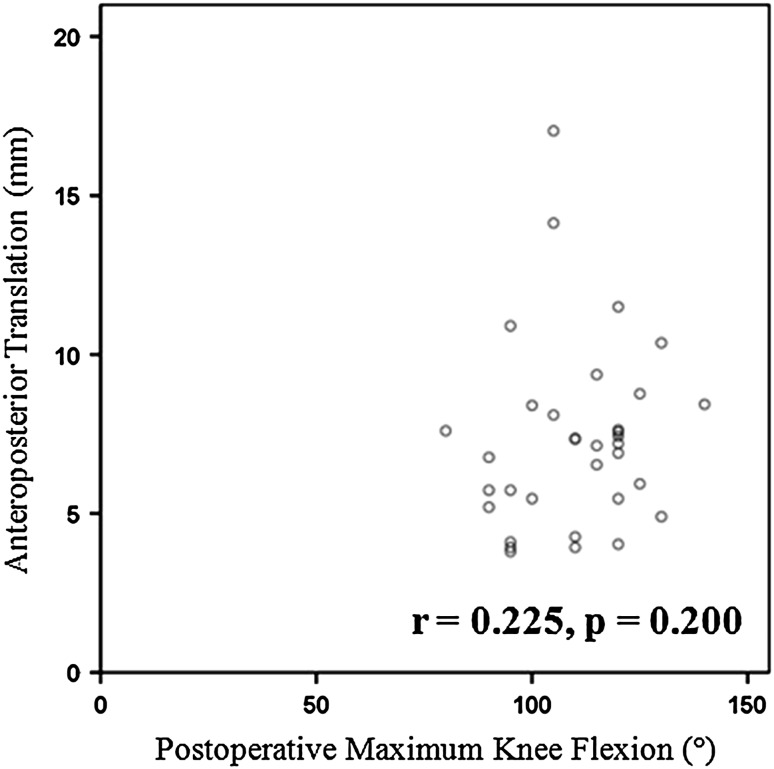
A graph shows no significant correlation between postoperative knee flexion and AP translation when the patient was awake.

There was no correlation between postoperative maximum knee flexion and AP translation in awake patients (r = 0.225; p = 0.200) (Fig. 2). Median AP translation in awake patients was 7.3 mm (25th percentile, 75th percentile: 5.5 mm, 8.3 mm).

**Fig. 3:**
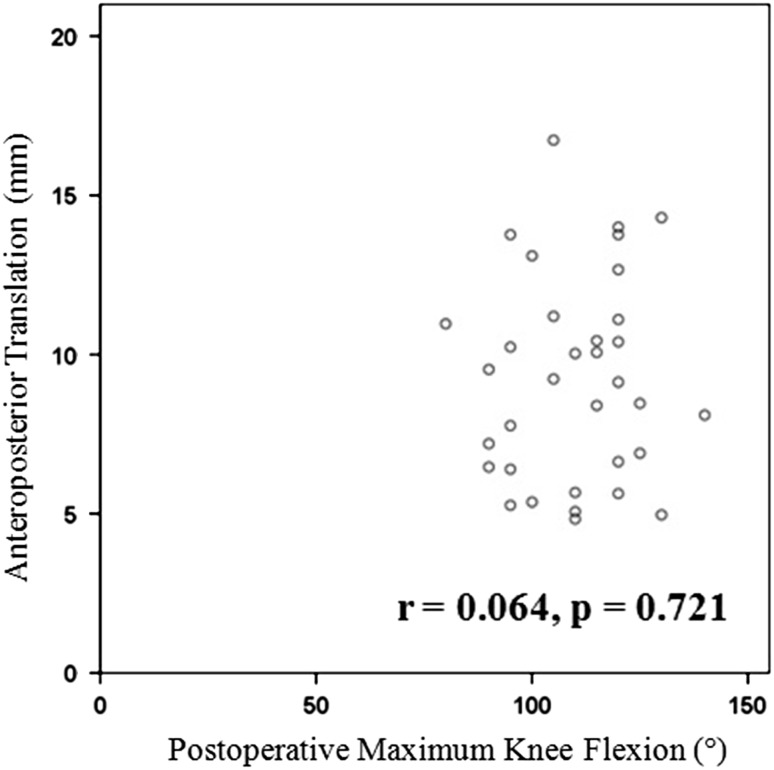
A graph shows no significant correlation between postoperative knee flexion and AP translation while the patient was under anesthesia.

There was no correlation between postoperative maximum knee flexion and AP translation in patients while under anesthesia (r = 0.064; p = 0.721) (Fig. 3). Median AP translation in anesthetized patients was 9.2 mm (25th percentile, 75th percentile: 6.5 mm, 11.1 mm).

**Fig. 4:**
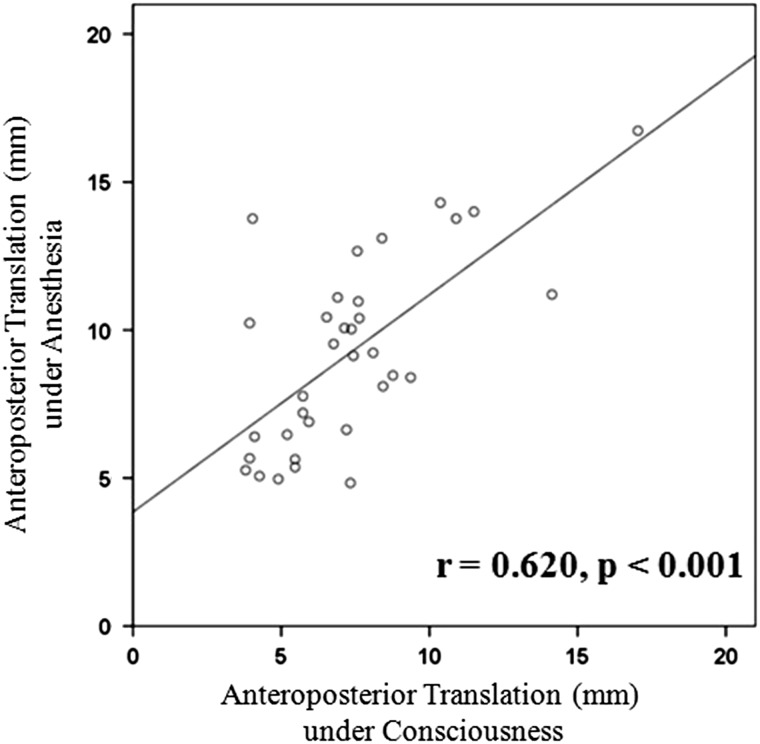
A graph shows a significant correlation of AP translation between consciousness and while the patient was under anesthesia.

Measurements of AP translation in the same patients in the awake and anesthetized conditions revealed significantly positive correlation (r = 0.620; p < 0.001) (Fig. 4). There was a significant difference in AP translation during consciousness versus under anesthesia (p < 0.001).

## Discussion

AP translation may be an important predictor of ROM after TKA [3, 8, 10, 21, 24, 26], and ROM after TKA is known to be a predictor of patient satisfaction with the procedure [12, 18, 19]. However, studies of AP translation in conscious individuals disagree regarding whether AP translation affects maximum knee flexion angle after TKA [3, 8, 10, 21, 24, 26]. We therefore sought to determine whether AP translation correlated with maximum knee flexion angle in patients who were (1) awake and (2) under anesthesia (to minimize the effects of voluntary muscle contraction) in a TKA with implantation of a PCL-substituting mobile-bearing prosthesis. We found that AP translation in the awake and in the anesthetized patient did not correlate with postoperative maximum knee flexion; that is, voluntary guarding of soft tissue structures did not change the result on this point. We also found that AP translation was greater in patients under anesthesia than in patients who were awake, but that measurements made in the same patient in the awake and anesthestized conditions were well correlated.

This study has some limitations. First, the results may not be generalized to all patients with knee arthroplasties because the study participants were patients with osteoarthritis who had well-balanced knees with few outliers for either ROM or AP translation after surgery. In addition, the sample size was relatively small although the numbers would be sufficient to detect a correlation coefficient of 0.5 by power analysis. Second, we did not investigate the effect of differences in geometry and/or soft tissue structures, such as a high-conformity design, post-cam design, or PCL-retaining design, on AP translation, because we intended to analyze only a PCL-substituting design. Previous studies [4, 6, 14, 24, 25] compared AP translation among different prostheses. In addition, the contributions of the supporting structures such as ACL, PCL, medial collateral ligament, and posterior capsules on AP displacement of the knee were described in a quantitative in vitro study [11]. Third, the interval between preoperative and postoperative knee flexion measurements varied between 6 and 114 months owing to study design, and this may have affected our results. However, we showed that with the current prosthetic design, knee ROM values after 24 months can be predicted statistically from the ROM values at 3 months [7]. Additionally, the average change in maximum knee flexion from 6 months to 3 years postoperatively was reported to be only 2.8° [20]. Moreover, we recognize that assessment of ROM under load-bearing conditions may provide better understanding of the factors influencing clinical performance during activity. Because of the characteristics of the study design, it was performed only under no axial load.

Fourth, we evaluated the AP translations at 75° flexion only. In retrospect, we should have evaluated the effects of soft tissue guarding at AP translation at 30° and 75° flexion using the same arthrometer. Iversen et al. [9] reported implications of muscular defense in testing for the anterior drawer sign in the knee at various angles in a stress radiograph investigation. They concluded that the opposing effect of the hamstrings on the anterior shift of the tibia was significantly less at 15° flexion than at 90°. Nevertheless, owing to the current prosthetic design characteristics, that is, constrained AP axis and full contact between the femoral component and the tibial insert from 0° to 30°, theoretically we would not expect any translation to occur at 30° knee flexion. Fifth, we should take the influence of voluntary and involuntary contractions into account in the awake evaluation, since we did not use EMG monitoring to confirm the degree of muscle relaxation. Finally, we evaluated only total AP translation. Although the arthrometer we used could measure anterior and posterior translation separately, the starting position of the femoral component in relation to the tibial component varied and was not easily identifiable. We recognize that a relatively posterior position of the femur on the tibia correlates significantly with maximum knee flexion [2]. However, the arthrometer we used is not only reliable and widely used to evaluate AP translation but also is noninvasive to the study participants. Despite the above limitations, a major strength of the study is that one experienced surgeon (YI), using the same instrumentation for all cases, treated all patients. Furthermore, the study provides unique information regarding the correlation between AP translation with and without anesthesia and the knee flexion angle necessary after TKA to overcome the effect of voluntary guarding of soft tissue structures.

A postoperative AP translation of approximately 5 to 10 mm is believed to be the preferred value for TKA using various arthrometers [4, 6, 10, 13, 14, 16, 24–26]. Some of these studies evaluated the correlation between AP translation and maximum knee flexion or ROM [3, 8, 10, 21, 24, 26] (Table 2).

**Table 2:** Preferred values for postoperative AP translation

Study	Implant design	PCL	AP translation
Chouteau et al. [3]	(Innex^®^ PCL retaining design	Retaining	12–13 mm
Itokazu et al. [8]	Miller-Galante^®^ PCL-retaining PCL-retaining design	Retaining	5.05 mm
Jones et al. [10]	PCA^®^ or Duracon^®^ prostheses	Retaining	5 to 10 mm
Seon et al. [21]	e.motion^®^ PCL retaining design	Retaining	7.1 mm
Warren et al. [24]	Insall-Burstein™ posterior stabilized knee	Substituting	Greater than 5 mm
	Kinemax^®^ condylar knee	Retaining	
	Oxford meniscal knee	Retaining	
Yamakado et al. [26]	Yoshino/Shoji-4 PCL-retaining design and Anatomic Graduated Components-Shoji PCL-retaining design	Retaining	9.71 mm

Innex^®^, Zimmer, Winterthur, Switzerland; Miller-Galante^®^, Zimmer, Warsaw, IN, USA; PCA^®^, Howmedica, Rutherford, NJ, USA; Duracon^®^, Howmedica, Rutherford, NJ, USA; e.motion^®^, Aesculap, Tuttlingen, Germany; Insall-Burstein™, Johnson & Johnson, New Milton, Hampshire, UK; Kinemax^®^, Howmedica International, Staines, Middlesex, UK; Oxford, Biomet Ltd, Swindon, Wiltshire, UK; Yoshino/Shoji-4,Biomet, Warsaw, IN, USA.

In our study, we did not find a correlation between AP translation in patients who were awake and maximum knee flexion. However, it is possible that outliers in terms of AP translation could affect knee flexion. Our study was small and because the knees generally were well balanced, the AP translation in patients who were awake averaged 7 mm (well within the desired range). We had few outliers in terms of AP translation, and therefore we might not have been able to detect an effect of incorrect AP translation on flexion if one were to have been present.

Although AP translation in patients under anesthesia was greater than it was in patients who were awake (by an average of approximately 2 mm), it was well correlated with the translation values in the patient who was awake, and it was no more predictive of maximum knee flexion than was AP translation in the patient who was awake. If the soft tissue conditions observed in patients under anesthesia in this study can be regarded as the same as those seen intraoperatively in patients during the index arthroplasty, surgeons should estimate that intraoperative AP translation is approximately 2 mm greater than what the patient will experience while awake.

We found that AP translation does not correlate with maximum postoperative knee flexion in patients under anesthesia or while awake. However, our results were obtained in patients whose TKAs generally were well balanced, with ranges of AP translation generally in the ideal range (approximately 5 to 10 mm) as described in other studies [4, 6, 10, 13, 14, 16, 23–26]. Our results should not be considered to mean that AP translation, or stability more generally, is unimportant. To the contrary, proper stability and balance of TKAs is important to ROM and function. In a previous study, Matsuda et al. [15] concluded that coronal stability, especially balanced stability, is important for achieving improved ROM in the same mobile-bearing design that was used in the current patients. These findings could support the importance of well-balanced stability to obtain maximum postoperative knee flexion after TKA.
